# Response rates in clinical quality registries and databases that collect patient reported outcome measures: a scoping review

**DOI:** 10.1186/s12955-023-02155-5

**Published:** 2023-07-11

**Authors:** Rasa Ruseckaite, Chethana Mudunna, Marisa Caruso, Susannah Ahern

**Affiliations:** School of Public Health and Preventive Medicine, Monash University, Melbourne, Victoria 3004 Australia

**Keywords:** Patient reported outcome measures, Surveys and questionnaires, Clinical registries, Response rates, Quality of life

## Abstract

**Background:**

Patient Reported Outcome Measures (PROMs) are being increasingly introduced in clinical registries, providing a personal perspective on the expectations and impact of treatment. The aim of this study was to describe response rates (RR) to PROMs in clinical registries and databases and to examine the trends over time, and how they change with the registry type, region and disease or condition captured.

**Methods:**

We conducted a scoping literature review of MEDLINE and EMBASE databases, in addition to Google Scholar and grey literature. All English studies on clinical registries capturing PROMs at one or more time points were included. Follow up time points were defined as follows: baseline (if available), < 1 year, 1 to < 2 years, 2 to < 5 years, 5 to < 10 years and 10 + years. Registries were grouped according to regions of the world and health conditions. Subgroup analyses were conducted to identify trends in RRs over time. These included calculating average RRs, standard deviation and change in RRs according to total follow up time.

**Results:**

The search strategy yielded 1,767 publications. Combined with 20 reports and four websites, a total of 141 sources were used in the data extraction and analysis process. Following the data extraction, 121 registries capturing PROMs were identified. The overall average RR at baseline started at 71% and decreased to 56% at 10 + year at follow up. The highest average baseline RR of 99% was observed in Asian registries and in registries capturing data on chronic conditions (85%). Overall, the average RR declined as follow up time increased.

**Conclusion:**

A large variation and downward trend in PROMs RRs was observed in most of the registries identified in our review. Formal recommendations are required for consistent collection, follow up and reporting of PROMs data in a registry setting to improve patient care and clinical practice. Further research studies are needed to determine acceptable RRs for PROMs captured in clinical registries.

**Supplementary Information:**

The online version contains supplementary material available at 10.1186/s12955-023-02155-5.

## Introduction

Clinical quality registries systematically monitor quality of healthcare within specific clinical domains by routinely collecting, analysing and reporting health-related information [1–4]. They use predefined set of indicators designed to assess variation across structural, process and outcome measures to benchmark quality of care. Registries have received increasing attention as a means of improving quality and reducing the cost of health and medical care, through identifying variations in clinical practice and assessing the uptake of effective treatment [4].

Patient reported outcome measures (PROMs) are standardized, validated questionnaires designed to assess patients’ perceptions of their own physical and mental status and wellbeing [5]. PROMs are increasingly being introduced in clinical registries, providing a personal perspective on the expectations and impact of treatment [6]. These instruments can complement the existing roles of registries and databases as platforms for quality assessment and benchmarking, as well as for large-scale research projects [6, 7]. PROMs are seen as useful information to reflect and improve on the clinical work undertaken by clinicians .

Including PROMs in clinical registries offers many advantages [6]. First, incorporating the patient voice ensures that measurement of healthcare outcomes is patient-centred. Second, symptom burden, health related quality of life (HRQoL) and satisfaction with care are essentially lost if not captured in “real time”. Third, capturing of comprehensive PROMs data in a registry setting can inform health service planning, research and evaluation, and facilitate benchmarking of participating health services.

PROMs offer an efficient and feasible way of incorporating the patient voice into healthcare outcome assessments and clinical decision-making. PROMs reporting and use for quality improvement is different for registries with regular patient contact and data collection over many years, compared to those registries capturing PROMs from few interactions. For the optimal utilisation, good quality data and high response rates (RRs) to PROMs are necessary [8]. In contrast to clinical outcomes, patient reported outcomes are self-reported, which inherently leads to concerns about RRs. RRs that reach 100% are hardly ever achieved, especially in routine chronic and advanced care [9, 10]. Although higher RRs have been considered desirable, the representativeness of PROMs samples in clinical registries has been rarely reported [11]. This has important practical implications with efforts required to succeed in implementing new routines and systematic collection of PROMs [12].

A recently conducted review of registry-based and cohort studies revealed a large variation in RRs to PROMs [13]. Although this review identified a large number of registries capturing PROMs, the registries examined were mainly from Scandinavia with the inclusion of only a few other registries from the UK and New Zealand. Further studies are needed to systematically evaluate trends in RRs across Europe, USA and other countries. The aim of the present study was to expand on this previous research and to identify from the existing literature as many as possible available clinical registries and databases with PROMs to describe their RRs and trends over time across various health conditions and world regions.

## Methods

### Protocol

The Arksey and O’Malley method and Preferred Reporting Items for Systematic Reviews and Meta-Analyses (PRISMA-ScR) procedures guided this review [14, 15]. The protocol was registered on PROSPERO (CRD42022344678).

### Information sources

To identify potential studies, a medical librarian searched two main electronic databases MEDLINE and EMBASE in collaboration with the primary author. Grey literature to identify registry websites and annual reports with the information on PROMs data collection and most recent RRs was also included. In addition, a list of Australian registries collecting PROMs was compiled via the website of the Australian Register of Clinical Registries (https://www.safetyandquality.gov.au/publications-and-resources/australian-register-clinical-registries).

### Eligibility criteria

Journal articles, annual reports and websites discussing registries or databases that collect PROMs data at one or more follow up time points and reporting PROMs RRs were included. Non-English articles, studies that did not use registry or database data and articles not reporting PROMs were excluded. Publications such as tutorials, letters, editorials, conference materials, periodical indices, personal narratives, practice guidelines or media were also excluded.

### Search strategy

The search strategy was adopted from Wang et al. [13] and modified to fit the scope of this study. We used Medical Subject Heading (MeSH) keywords and free text search terms. The database records and details of how the literature search was undertaken was maintained at each stage of the review process. The terms were combined by means of Boolean operators and are listed in Additional file 1. A manual search of grey literature was performed. All searches were performed in August 2022.

### Study selection

For each article selected for inclusion, abstracts and full texts were obtained. Reference lists of the included studies and systematic reviews were examined during the initial review.

The titles and abstracts of journal articles were screened by two researchers (CM and MC). Both authors then read the full texts of these articles to assess eligibility for final inclusion. Disagreement between the authors regarding eligibility was resolved by consensus amongst the three authors (CM, MC and RR). The inclusion and exclusion criteria were applied once again, and articles meeting the inclusion criteria progressed to the next stage of the review for data extraction. All screening processes were conducted through Endnote X9.

In the third phase, two independent researchers (CM and MC) extracted data from the eligible studies into a standardized excel spreadsheet. All discrepancies during the review process were resolved and verified by the lead researcher RR [15].

### Data management

Relevant data from the included articles were extracted by CM, MC and RR. Data from grey literature such as registry annual reports and registry websites were also extracted by the same researchers during the data extraction phase. Data extracted from the journal articles, reports and websites included: country, registry name, source of information, condition, year registry was established, year registry started collecting PROMs data, number of patients in the registry, PROMs captured, number of reminders sent, RRs at various follow up time points, and any other relevant information. Methods used to calculate RRs were not explicitly stated in most articles, reports and websites, therefore this information was not included. If relevant information could not be located, an email to the registry contact was sent with a request for the missing information.

The extracted data was synthesized according to three steps: (1) analysing the data, (2) reporting the findings, (3) discussing the implications [15].

### Data analysis and statistics

PROMs RRs from each registry and database were grouped according to the follow up time points of data collection. Follow up time points were defined as follows - t0: baseline (where available), t1: 0 to 1 year, t2 : 1 to < 2 years, t3 : 2 to < 5 years, t4 : 5 to < 10 years, and t5 : 10 + years. Registries were further grouped into the regions of the world: North and South American, European (excluding Scandinavia), Scandinavian, Oceania (including Australia and New Zealand), Asian and Global (those covering all continents). They were also categorised according to health conditions they captured: Arthroplasty/Reconstruction/joint related procedures, Chronic disease, Cancer, Trauma/Burns/Pain, Spine, Cardiac, Rare disease, Gynaecological, General surgery and device, and Miscellaneous conditions.

Change in RRs was calculated by subtracting the final reported RR from baseline or first reported RR and dividing the difference by the total length of follow up time. Registries that reported RR at a single follow up time were excluded from these calculations.

## Results

### General description of the literature

**Fig. 1:**
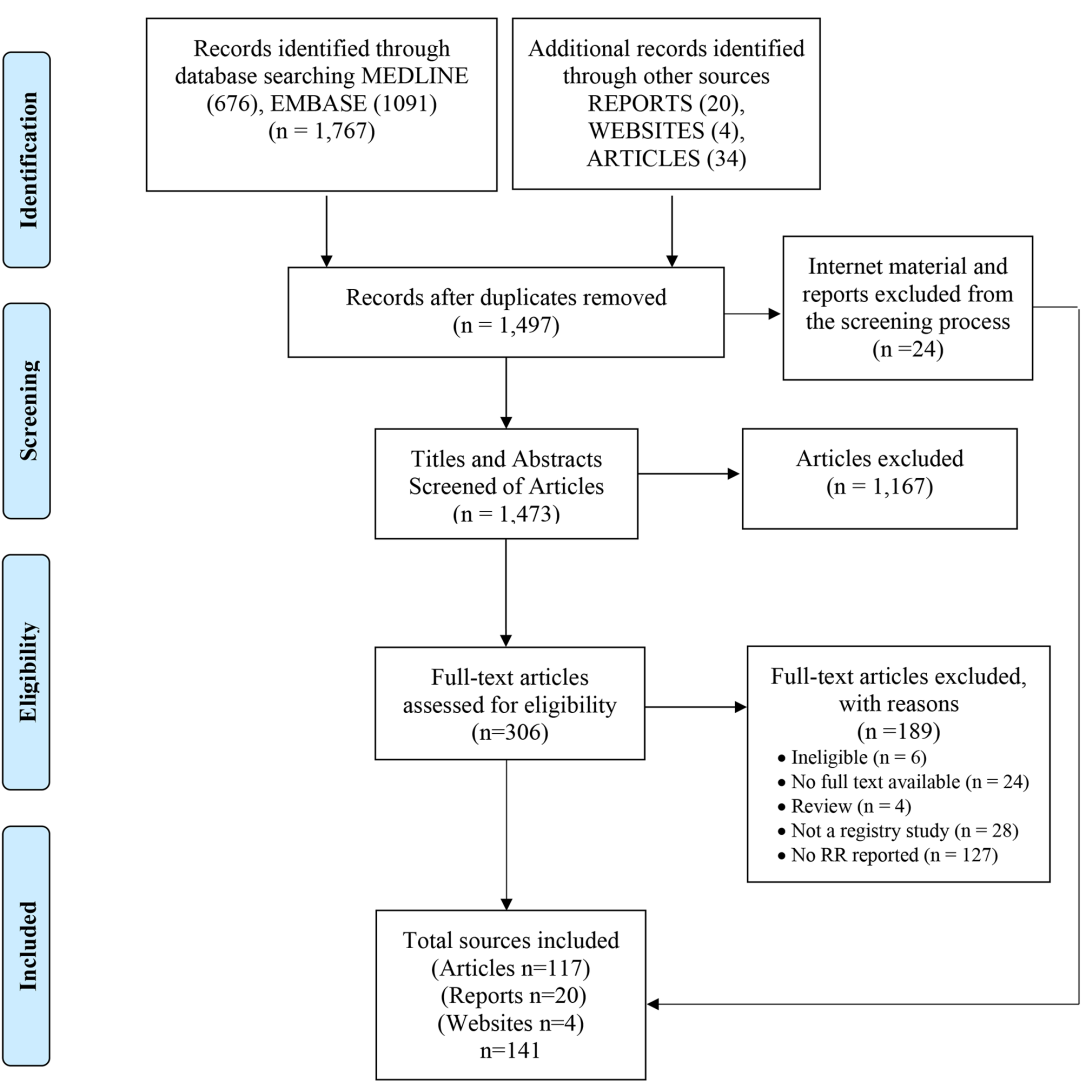
PRISMA chart

The search strategy yielded 1,767 publications (Fig. 1). A further 58 citations including grey literature and websites were identified. After removing duplicates, 1,497 sources remained. Twenty-four internet materials were excluded from the initial article screening process. Titles and abstracts of 1,473 journal articles were screened according to the inclusion criteria. Of those, 306 full text articles were assed for eligibility. The screening of full texts resulted in 117 journal articles. Combined with the 20 reports and 4 websites, a total of 141 sources were used in the data extraction and analysis process.

Articles in this review were published between 2008 and 2022. Twelve (8.5%) publications were published in 2022, 31 (21.9%) articles were published in 2021, 28 (19.9%) in 2020, 22 (15.6%) in 2019 and the remaining articles were published between the years 2008 and 2018 (Table 1).

**Table 1:** Registry characteristics. Follow up point t0 is the reported baseline time point or time of intervention as specified in the article or report. Follow up point t1 is from 0 to 1 year, follow up point t2 is from 1 to < 2 years, follow up point t3 is from 2 to < 5 years, follow up point t4 is from 5 to < 10 years and follow up point t5 is from 10 + years

Source of Information	Registry	Condition	Country	Mode of Administration	No of reminders	RR (%) at follow up points						Total follow up (years)	Change in RR (%/year)
						t0	t1	t2	t3	t4	t5		
Annual Report (2021) [41]	AOANJRR	Arthroplasty	Australia	Electronic	1	66.7	61.5	NA	NA	NA	NA	0.5	-10.5
Heo et al. (2019) [42], Churches et al. (2018) [43]	ACORN	Arthroplasty	Australia	Paper, phone	7	86.4	74	NA	NA	NA	NA	NA	NA
Rolfson et al. (2011) [44]	WRHA Joint Replacement	Arthroplasty	Canada	Paper	0	75	NA	NA	NA	NA	NA	NA	NA
Annual Report (2020–2021) [45]	VOTOR	Arthroplasty	Australia	Phone	NS	85	80	76	NA	NA	NA	2	-4.5
Annual Report (2022) [46]	Australian & New Zealand Hip Fracture Registry	Arthroplasty	Australia & New Zealand	NA	NA	NA	53	NA	NA	NA	NA	1	-12
Annual Report 2021–2022 [41]	Sydney Orthopaedic Foot & Ankle Research Institute Clinical Quality	Arthroplasty	Australia	NA	NA	78	66	NA	NA	NA	NA	0.33	NA
Scholes et al. (2023) [47]	PRULO	Arthroplasty	Australia	NA	NA	72	NA	NA	NA	NA	NA	NA	NA
Galea et al. (2020) [48]	DHAR	Arthroplasty	Denmark	Electronic	2	70	62	68	68	NA	NA	2	-1
Mechlenburg et al. (2020) [49]	DSR	Arthroplasty	Denmark	Electronic, paper	NS	93	NA	62	NA	NA	NA	1	-31
Rolfson et al. (2016) [50]	RIPO	Arthroplasty	Italy	Electronic	NS	30	NA	80	NA	NA	NA	1	50
Rolfson et al. (2016) [50]	LAR	Arthroplasty	Lithuania	Phone	0	100	60	NA	NA	NA	NA	0.5	-80
Most et al. (2022) [51]	LROI	Arthroplasty	Netherlands	Electronic, paper	0	38	50	38	NA	NA	NA	1	0
Rolfson et al. (2016) [50], Annual Report (2021) [24]	NZACL	Arthroplasty	New Zealand	Electronic, paper	> 1	100	86	80	86	NA	NA	2	-7
Rolfson et al. (2016) [50], Annual Report (2021) [52]	NZJR	Arthroplasty	New Zealand	Paper	0	NA	72.5	NA	NA	NA	NA	0.5	NA
Annual Report (2022) [53]	NAR	Arthroplasty	Norway	NS	NS	98	NA	80	NA	NA	NA	1	-18
Bartels et al. (2018) [54], Kristensen et al. (2017) [55], Gjertson et al. (2008) [56], Annual Report (2021) [57]	NHFR	Arthroplasty	Norway	Paper	0	28.2	57	53	NA	NA	NA	1	24.8
Ulstein et al. (2018 ) [58], Annual Report (2021) [53]	NKLR	Arthroplasty	Norway	Paper	1	NA	NA	NA	57.5	52.1	52	10	NA
Goh et al. (2021) [59]	Singapore Institutional Joint Registry	Arthroplasty	Singapore	NS	NS	100	89	NA	72	NA	NA	2	-14
SAR Annual Report (2021) [60]	SAR	Arthroplasty	Sweden	Electronic, paper	1	85.5	NA	82.5	NA	NA	NA	1	-3
Ulstein et al. (2018) [58], Annual Report (2019) [61]	Swedish Knee Ligament Register	Arthroplasty	Sweden	Electronic, paper	1	60	NA	55	50	45	40	10	-2
Coster et al. (2020) [62]	Swefoot	Arthroplasty	Sweden	Electronic, Paper	1	75	NA	72	NA	NA	NA	1	-3
Joelson et al. (2021) [63]	Swespine	Arthroplasty	Sweden	Electronic, Paper	NS	94	NA	75	65	NA	NA	2	-14.5
Kamrad et al. (2017) [64]	SwedAnkle	Arthroplasty	Sweden	Paper	NS	NA	55	76	30	NA	NA	2	NA
Rolfson et al. (2016) [50], Annual Report (2010) [65]	Geneva Hip Arthroplasty Registry	Arthroplasty	Switzerland	Electronic, paper, phone	2	77	NA	65	NA	76.9	70.8	10	-0.6
Maempel (2018) [66], Annual Report (2022) [67]	NAHR	Arthroplasty	UK	Electronic, paper, phone	2	96.5	50	40	7.5	NA	NA	2	-44.5
Annual Report (2021) [68]	UK National Joint Registry	Arthroplasty	UK	Electronic, paper	0	51.7	35	NA	11%	29.9	NA	5	-4.4
Annual Report (2015) [69]	UK NLR	Arthroplasty	UK	Electronic, paper	> 1	61.3	47	42	33	NA	NA	2	-14.1
Patel et al. (2015) [70]	CJRR (California)	Arthroplasty	USA	Electronic, paper, phone	NS	30.2	10	18.1	NA	NA	NA	1	-11.9
Randsborg et al. (2022) [71]	HSS ACL Registry	Arthroplasty	USA	NS	NS	31	NA	NA	31	51.2	NA	7.2	2.8
Lyman (2018) [72]	HSS Joint Replacement Registry	Arthroplasty	USA	Electronic	NS	80	NA	NA	81	NA	NA	2	0.5
Annual Report (2021) [73]	AJRR	Arthroplasty	USA	Electronic, paper	NS	25	NA	27.8	NA	NA	NA	1	2.8
Rolfson et al. (2016) [50]	FORCE TJR	Arthroplasty	USA	NS	NS	82.5	82.5	NA	NA	NA	NA	0.5	0
Rolfson et al. (2016) [50]	Michigan Arthroplasty Registry	Arthroplasty	USA	NS	NS	32	12	NA	NA	NA	NA	1	-20
Annual Report (2020) [74]	PCOR-ANZ	Cancer	Australia	Electronic, paper, phone	NS	57	NA	57%	NA	NA	NA	1	0
Rechtman et al. (2022) [29], Annual Report (2020) [74]	PCOR-VIC	Cancer	Australia	Electronic, paper, phone	> 1	98.5	NA	75	75	NA	NA	2	-11.8
Annual Report (2020) [74]	PCOR-QLD	Cancer	Australia	Paper	NS	NA	49	NA	NA	NA	NA	1	NA
Annual Report (2020) [74]	PCOR-ACT	Cancer	Australia	Electronic, paper, phone	NS	NA	62	NA	NA	NA	NA	1	NA
Annual Report (2020) [74]	PCOR-NSW	Cancer	Australia	Electronic, paper, phone	NS	NA	48	NA	NA	NA	NA	1	NA
Annual Report (2020) [74]	PCOR-NT	Cancer	Australia	Paper	NS	NA	39	NA	NA	NA	NA	1	NA
Annual Report (2020) [74]	PCOR-NZ	Cancer	Australia	Electronic, paper	NS	NA	68	NA	NA	NA	NA	1	NA
Annual Report (2020) [74]	PCOR-SA	Cancer	Australia	Paper	NS	NA	33	NA	NA	NA	NA	1	NA
Annual Report (2020) [74]	PCOR-TAS	Cancer	Australia	Electronic, paper, phone	NS	NA	44	NA	NA	NA	NA	1	NA
Ettridge et al. (2021) [75]	SAPCCOC Registry	Cancer	Australia	Paper	NS	NA	75	60	57	NA	NA	2	NA
Skandarajah et al. (2021) [76]	VCR	Cancer	Australia	Paper	1	45.6	NA	47.3	48.1	41.5	NA	5	-0.8
Sztankay et al. (2019) [27]	aMYELOIDr	Cancer	Austria	Electronic	NS	99	94	NA	NA	NA	NA	NA	NA
Vasquez et al. (2020) [77]	APCaRI Registry & Biorepository	Cancer	Canada	Electronic, paper	NS	77	NA	NA	NA	NA	NA	NA	NA
Joachim et al. (2018) [78]	MCR	Cancer	Caribbean	Electronic, paper	1	85	NA	NS	NS	70	NA	5	NA
Rose et al. (2020) [79]	DBCG Registry	Cancer	Denmark	Electronic	1	60	NA	NA	NA	48.3	NA	5	-2.3
Bronserud et al. (2019) [80]	DLCR	Cancer	Denmark	Electronic, paper	2	NA	50.4	48.9	NA	NA	NA	1	NA
Nguyen-Nielsen et al. (2016) [81]	DAPROCAdata	Cancer	Denmark	Electronic, paper	NS	26	NA	92	NA	NA	NA	1	66
Wallwiener et al. (2017) [82]	PRAEGNANT Register	Cancer	Germany	Electronic	0	57	33	NA	NA	NA	NA	0.5	0
Lovegrove et al. (2020) [83]	HEAT Registry	Cancer	Global	Electronic	NS	72.6	25.4	NA	NA	NA	NA	NA	NA
Gupta et al. (2021) [84]	PanCAN	Cancer	Global	Electronic	NS	77.6	NA	75	NA	NA	NA	1	-2.6
Van Kleef et al. (2021) [85]	NCR	Cancer	Netherlands	Electronic, paper	0	85	14.8	NA	NA	NA	NA	0.02	[Outlier]
Ramsey et al. (2019) [86]	PROFILES Registry	Cancer	Netherlands	Electronic, paper	1	73	NA	83	81.3	NA	NA	4	2
Christiansen et al. (2019) [87]	CRN	Cancer	Norway	Electronic	1	97	91	NA	NA	NA	NA	0.3	-24
Amit et al. (2019) [88]	Oropharynx Cancer Registry PROF Core	Cancer	USA	Paper	1	84	NA	NA	NA	NA	NA	NA	NA
Hoffman et al. (2020) [23]	CaPSURE	Cancer	USA	Paper	NS	100	97	94	85	77	NA	5	-4.6
Barker et al. (2018) [89], Annual Report (2020) [90]	VCOR	Cardiac	Australia	Electronic, phone	2	NA	72	NA	NA	NA	NA	0.08	NA
Ikemura et al. (2019) [25]	KiCS-AF registry (Cohort Study)	Cardiac	Japan	Electronic, paper	NS	97	NA	91.7	NA	NA	NA	1	-5.3
Steingerg et al. (2020) [91], Piccini et al. (2011 ) [28]	ORBIT-AF	Cardiac	USA	Electronic	NS	94	80	67	NA	NA	NA	1	-27
Bradley et al. (2019) [92]	PALM Registry	Cardiac	USA	Electronic	0	93	NA	NA	NA	NA	NA	NA	NA
Arnold et al. (2022) [93]	STS, ACC & TVT Registry	Cardiac	USA	NS	NS	25	25	NA	NA	NA	NA	0.08	0
Annual report (2020–2021) [94]	VACAR	Cardiac	Australia	Phone	3	NA	85	NA	NA	NA	NA	1	NA
Alarcon et al. (2020) [22]	COREXH Registry	Chronic Disease	Columbia	Paper	NS	99	81	64	NA	NA	NA	1	-35
Apfelbacher et al. (2019) [95]	CARPE	Chronic Disease	Germany	Electronic, paper	NS	NA	87	69	49	22	NA	NA	NA
Kreuter et al. (2017) [96]	INSIGHTS-IPF Registry	Chronic Disease	Germany	Electronic	NS	84.5	84.5	NA	NA	NA	NA	1	NA
Younossi et al. (2021) [97], Hardy (2020) [98]	Global NASH & NAFLD Registry	Chronic Disease	Global	Electronic	NS	50	NA	14	NA	NA	NA	0.3	-96
Ahmed et al. (2015) [99]	CARE	Chronic Disease	Netherlands	Electronic, paper	1	90	85	NA	82	NA	NA	2	-4
Verket et al. (2018) [100]	ORAR	Chronic Disease	Norway	Paper	0	71	NA	NA	75	59	61	15	-0.7
Nimmo et al. (2018) [9]	SRR	Chronic Disease	Scotland	Paper	0	NA	44	NA	NA	NA	NA	NA	NA
Mellgren et al. (2020) [101]	InfCareHiv	Chronic Disease	Sweden	Electronic, paper	0	NA	NA	44	NA	NA	NA	1	NA
Svedbo (2018) [102]	Swedish NDR	Chronic Disease	Sweden	Paper	1	NA	61	NA	NA	NA	NA	NA	NA
Hejike et al. (2020) [103], Hejike et al. (2021) [104]	KLURING	Chronic Disease	Sweden	NS	NS	100	76	90	72	49	NA	5	-10.2
Hofstedt et al. (2019) [105]	SRQ	Chronic Disease	Sweden	Electronic, paper	0	NA	88	NA	NA	NA	NA	NA	NA
Twigg et al. (2017) [106]	YEAR	Chronic Disease	UK	Paper	NS	92	74	74	NA	NA	NA	1	-18.5
Callis Duffin et al. (2021) [107], Mease et al. (2017) [108], Strober et al. (2019) [109]	Corrona PsA, SpA Registry	Chronic Disease	USA	Paper	NS	NA	99	NA	NA	NA	NA	NA	NA
Yun et al. (2020) [110]	Arthritis Power Registry	Chronic Disease	USA	Electronic	NS	NA	33	NA	NA	NA	NA	NA	NA
Case et al. (2020) [111]	IPF-PRO Registry	Chronic Disease	USA	NS	NS	76	NA	NA	NA	NA	NA	NA	NA
Braaten et al. (2019) [112]	UPI Arthritis Registry	Chronic Disease	USA	NS	NS	NA	58	NA	NA	NA	NA	NA	NA
Feldon et al. (2017) [113]	MYOVISION	Chronic Disease	USA & Canada	Electronic, paper, phone	1	91	NA	NA	NA	NA	NA	NA	NA
Zimmerman et al. (2019) [114]	Swedish National Quality Registry for Hand Surgery	General Surgery	Sweden	Electronic, paper	0	33	27	24	NA	NA	NA	1	-9
Hallenstal et al. (2021) [115]	NTSRS	General Surgery	Sweden	Electronic	0	NA	44	NA	NA	NA	NA	0.5	NA
Alvarez et al. (2021) [116], Waljee et al. (2015) [117]	MBSC	General Surgery	USA	Electronic	3	36	36	NA	NA	NA	NA	1	0
Helsten et al. (2016) [118]	SATISFY-SOS Registry	General Surgery	USA	Electronic, paper, phone	2	NA	62	71	NA	NA	NA	1	NA
Verket et al. (2018) [100]	Norwegian Endometriosis Association	Gynaecological Surgery	Norway	Paper	0	NA	25	25	NA	NA	NA	NA	NA
Annual Report 2021 [119]	ABDR	General Surgery	Australia	NS	NS	NA	40	40	38	NA	NA	5	-0.2
Melkemichel (2020) [120], Lundstrom et al. (2018) [121], Jakobsson et al. (2022) [122]	SHR	Gynaecological Surgery	Sweden	Paper	1	NA	NA	71	NA	NA	NA	1	NA
Madsen et al. (2017) [123], Nussler et al. (2022) [124]	GynOp	Gynaecological Surgery	Sweden	Paper	0	NA	90	85	NA	NA	NA	1	NA
Bradley et al. (2021) [125]	PFDR-R	Gynaecological Surgery	USA	Electronic, paper	NS	49	NA	NA	NA	NA	NA	NA	NA
Poulsen et al. (2018) [126]	National Patient Register	Miscellaneous	Denmark	Paper	1	NA	79	NA	NA	NA	NA	NA	NA
Wall et al. (2020) [127], Vuillermin (2021) [128]	CoULD Registry	Miscellaneous	Global	Electronic	0	NA	97	NA	NA	NA	NA	NA	NA
Ruiter et al. (2021) [129]	Dutch-Belgian Registry for NMJ Disorders	Miscellaneous	Netherlands & Belgium	Electronic	0	88	49	NA	NA	NA	NA	NA	NA
Moller et al. (2022) [130], Lagergren et al. (2020) [131], Juto et al. (2017) [132]	SFR	Miscellaneous	Sweden	Paper	1	55	NA	41	NA	NA	NA	1	-14
Lutz et al. (2020) [133]	ATI Patient Outcomes Registry	Miscellaneous	USA	NS	NS	54	NA	NA	NA	NA	NA	NA	NA
Pearl et al. (2020) [21]	ARMR	Miscellaneous	USA	Paper	0	99.5	NA	NA	NA	NA	NA	NA	NA
Annual Report (2020–2021) [134]	ADNeT	Miscellaneous	Australia	Paper	1	53	NA	NA	NA	NA	NA	NA	NA
Seefried et al. (2020) [20]	Global HPP Registry	Rare disease	Global	Electronic	NS	68	NA	NA	NA	NA	NA	NA	NA
Morris et al. (2018) [19]	FSHD Patient Registry	Rare disease	UK	Electronic	1	NA	89	NA	NA	NA	NA	NA	NA
Tosi et al. (2019) [16]	BBDC Contact Registry	Rare disease	USA	Electronic	NS	NA	87	NA	NA	NA	NA	NA	NA
Eng et al. (2021) [18]	EBCare Registry	Rare disease	USA	Electronic	0	19.5	NA	NA	NA	NA	NA	NA	NA
Annual Report (2022) [135]	ASR	Spine	Australia	Electronic, phone	3	85	82	83	78	NA	NA	2	-3.5
Andersen et al. (2018) [136]	DaneSpine	Spine	Denmark	Paper	1	90	NA	86	NA	NA	NA	1	-4.5
Matsumoto et al. (2021) [137]	PSSG & GSSG	Spine	Global	Electronic, paper	0	42	NA	43	64.9	NA	NA	2	11.5
Mannion et al. (2018) [138], Morris et al. (2018) [19], Sunderland et al. (2021) [139]	EUROSPINE Spine Tango Registry	Spine	Global	Electronic, paper	1	49	66	73.3	54	NA	NA	2	2.5
Austevoll et al. (2019) [140]	NORspine	Spine	Norway	Paper	0	NA	79	81	NA	NA	NA	1	NA
Zakaria et al. (2019) [141]	MSSIC	Spine	USA	Electronic, paper, phone	2	72	55	49	NA	NA	NA	1	-23
Theisen et al. (2020) [142]	NBRG SCI Registry	Spine	USA	NS	NS	92	NA	NA	NA	NA	NA	NA	NA
Wilkerson et al. (2022) [143]	NeuroPoint QOD	Spine	USA	NS	NS	67	82	70	NA	NA	NA	1	2.6
Annual Report (2020) [144]	AuSCR	Stroke	Australia	Paper, phone	2	92	65	NA	NA	NA	NA	0.5	-54
Palmcrantz et al. (2018) [145]	RisStroke	Stroke	Sweden	Paper, phone	0	NA	81	79	NA	NA	NA	1	NA
Turner et al. (2019) [146], Annual Report (2020–2021) [147]	BRANZ	Trauma	ANZ	Paper, phone	4	71	55	40	21	NA	NA	2	-25
Turner et al. (2019) [146]	VSTR	Trauma	Australia	Phone	NS	NA	87	88	86	NA	NA	2	NA
Stamer et al. (2021) [148], Zaslansky et al. (2015) [149]	PAIN OUT Infant	Trauma	Global	Electronic	NS	20.6	NA	NA	NA	NA	NA	NA	NA
Van der Vliet et al. (2019) [150]	DNTR	Trauma	Netherlands	Paper, phone	2	NA	75	98	NA	NA	NA	1	23
Wihlke et al. (2021) [151]	SweTrau	Trauma	Sweden	Paper, phone	1	NA	78	68	NA	NA	NA	1	NA
Kallman et al. (2020) [26]	SQRP	Trauma	Sweden	Electronic, paper	NS	99	86	55	NA	NA	NA	1	-44
Turner et al. (2019) [146]	TARN	Trauma	UK	Paper	NS	61	88	NA	NA	NA	NA	NA	NA
Rolfson et al. (2011) [44]	CERTs Registry	Trauma	USA	NS	NS	80	NA	NA	NA	NA	NA	NA	NA
Turner et al. (2019) [146], Rios-Diaz et al. (2017) [152]	FORTE project	Trauma	USA	Phone	NS	NA	45	42	NA	NA	NA	1	NA
Amtman et al. (2017) [153]	NIDILRR BMS National Database	Trauma	USA	Electronic, paper, phone	NS	82	72	63	54	NA	NA	2	-14

ABDR: The Australian Breast Device Registry, ACC: American College of Cardiology, ACL: Anterior Cruciate Ligament, ACORN: Arthroplasty Clinical Outcomes Registry, National,ADNet: Australian Dementia Network Registry, AJRR: American Joint Replacement Registry, aMYELOIDr: Austrian Myelome Registry, AOANJRR: Australian Orthopaedic Association National Joint Replacement Registry, APCaRI: Alberta Prostate Cancer Research Initiative, ARMR: American Registry for Migraine Research ASR: Australian Spine Registry, AuSCR: Australian Stroke Registry, BBDC: Brittle Bone Disorders Consortium, BMS: Burn Model System, BRANZ: The Burns Registry of Australia and New Zealand, CaPSURE: Prostate Strategic Urologic Research Endeavor prostate cancer registry, CARE: Dutch Chronic Pancreatitis Registry, CERTs: Centre for Education and Research on Therapeutics, COREXH: Expanded Haemodialysis Registry Protocol in Colombia, CoULD: The Congenital Upper Limb Difference, CRN: Cancer Registry in Norway, DaneSpine: Danish National Spine Database, DAPROCA data: Danish Prostate Cancer Database, DBCG: Danish Breast Cancer Cooperative Group, DHAR: Danish Hip Arthroplasty Registry, DLCR: Danish Lung Cancer Registry, DNTR: Dutch National Trauma Registry, EB: Epidermolysis Bullosa, DSR: Danish Shoulder Arthroplasty Registry; FORTE: Functional Outcomes and Recovery after Trauma Emergencies, FSHD: Facioscapulohumeral Dystrophy, GSSG: Growing Spine Study Group, GynOp: Swedish National Quality Register of Gynaecological Surgery, Heat: High Intensity Focused Ultrasound Evaluation and Assessment of Treatment, HPP: Hypophosphatasia, HSS: Hospital for Special Surgery, HSS: Hospital for Special Surgery, IPF-PRO: The Idiopathic Pulmonary Fibrosis Prospective Outcomes, KiCS- AF: Keio Interhospital Cardiovascular Studies-atrial fibrillation, KLURING: Clinical Lupus Register in North-Eastern Gothia, Sweden, LAR: Lithuanian Arthroplasty Registry, LROI: Dutch Arthroplasty Register, MBSC: Michigan Bariatric Surgery Collaborative, MCR: Martinique Cancer Registry, MSSIC: Michigan Spine Surgery Improvement Collaborative, NAFLD: Non-Alcoholic Fatty Liver Disease, NAHR: British Non-Arthroplasty Hip Register, NAR: Norwegian Arthroplasty Register, NASH: Non-Alcoholic Steatohepatitis, NBRG: Neurogenic Bladder Research Group, NCR: Netherlands Cancer Registry, NDR: National Diabetes Register, NHFR: Norwegian Hip Fracture Register, NIDILRR: National Institute on Disability, Independent Living, and Rehabilitation Research, NJM: Neuromuscular Junction, NKLR: Norwegian Knee Ligament Register, NLR: National Ligament Register, NORspine: Norwegian National Spine Registry, NTSRS: National Tonsil Surgery Register in Sweden, NZACL: New Zealand Anterior Cruciate Ligament Registry, NZJR: New Zealand Joint Registry, ORAR: Oslo Rheumatoid Arthritis Register, ORBIT-AF: Outcomes Registry for Better Informed Treatment of Atrial Fibrillation, PALM: Patient and Provider Assessment of Lipid Management, PanCAN: The Pancreatic Cancer Action Network, PCOR ACT: Prostate Cancer Outcomes Registry - Australian Capital Territory, PCOR-ANZ: Prostate Cancer Outcomes Registry - Australia and New Zealand, PCOR- NSW: Prostate Cancer Outcomes Registry – New South Wales, PCOR- NT: Prostate Cancer Outcomes Registry – Northern Territory, PCOR NZ: Prostate Cancer Outcomes Registry New Zealand, PCOR- QLD: Prostate Cancer Outcomes Registry - Queensland, PCOR SA: Prostate Cancer Outcomes Registry South Australia, PCOR TAS: Prostate Cancer Outcomes Registry - Tasmania, PCOR-VIC: Prostate Cancer Outcomes Registry Victoria, PFD-R: American Urogynecology Society’s Pelvic Floor Disorder Registry for Research, PROF: Patient Reported Outcomes and Function, PROF: Patient Reported Outcomes and Function, PsA: Psoriatic Arthritis, PSSG: Paediatric Spine Study Group, PRULO; Patient Registry of Upper Limb pathology Outcome, QOD: Quality Outcomes Database, RIPO: Register of the Orthopaedic Prosthetic Implants, RisStroke: Swedish National Stroke Register, SAPCCOC: South Australian Prostate Cancer Clinical Outcomes Collaborative, SAR: Swedish Arthroplasty Registry, SATISFY-SOS: Systematic Assessment and Targeted Improvement of Services Following Yearlong Surgical Outcomes Survey, SCI: Spinal Cord Injury, SFR: Swedish Fracture Register, SHR: Swedish Hernia Register, SpA: Spondyloarthritis, SQR: Swedish Rheumatology Quality Register, SQRP: Swedish Quality Registry for Pain Rehabilitation, SRR: Scottish Renal Registry, STS: Society of Thoracic Surgeons, SwedAnkle: The Swedish Ankle Registry, Swespine: Swedish National Spine Register, SweTrau: Swedish Trauma Register, TARN: Trauma Audit and Research Network, TVT :Transcatheter Valve Therapy, UK: United Kingdom, UPI: Utah Psoriasis Initiative, VACAR; Victorian Ambulance Cardiac Arrest Registry, VCOR: Victorian Cardiac Outcomes Registry, VCR: Victorian Cancer Registry, VOTOR: Victorian Orthopaedic Trauma Outcomes Registry, VSTR: Victorian State Trauma Registry, WRHA: Winnipeg Regional Health Authority, YEAR: Yorkshire Early Arthritis Register

Following the data extraction, 121 registries were identified and included for evaluation of RRs. Of the 121 registries, 33 (27%) were located in North and South America. Thirty-one (26%) registries originated from Scandinavia and 20 (17%) were based elsewhere in Europe. Twenty-three (19%) registries were located in Australia and New Zealand. The remaining eight (7%) registries were classified as global.

Arthroplasty/Reconstruction/Joint related procedure registries (27%) were most frequently reported in the literature. Cancer registries accounted for 21%, followed by 16%for chronic disease registries.

Twenty-five (21%) registries exclusively collected PROMs electronically (Table 1). Twenty-eight (23%) registries captured PROMs on paper. Forty-seven (39%) registries used a combination method for collecting PROMs, and only three (4%) exclusively phoned their patients to capture PROMs.

Information on PROMs reminders was available for 63 (52%) registries. Twenty-four (20%) registries did not send any reminders, 22 (18%) sent one reminder while 17 (14%) registries sent more than one reminder.

### Registries collecting PROMs at various follow up time points

The vast majority (76%) of registries captured PROMs data at baseline (Table 2). In North and South America, baseline PROMs were captured by 27 (82%) registries, followed by 24 (77%) in Scandinavia, 18 (90%) in other European countries and 14 (61%) registries in Oceania. PROMs at < 1 year follow up were captured by 14 (42%) North and South American registries, 13 (42%) Scandinavian and 13 (65%) for both European and Oceania registries. Similarly, 21 (68%) Scandinavian registries, 17 (54%) North and South American registries, 17 (74%) Oceania and 6 (30%) European registries captured PROMs at 1 to < 2 years follow up. These numbers decreased with follow up years.

**Table 2:** Number and proportion of registries collecting PROMs at various follow up time points stratified by region and condition. Follow up point t0 is the reported baseline time point or time of intervention as specified in the article or report. Follow up point t1 is from 0 to 1 year, follow up point t2 is from 1 to < 2 years, follow up point t3 is from 2 to < 5 years, follow up point t4 is from 5 to < 10 years and follow up point t5 is from 10 + years

Registry type	Follow up points					
	t0	t1	t2	t3	t4	t5
**All registries (n = 121)**	92 (76%)	57(47%)	69 (57%)	33 (27%)	12 (10%)	2 (2%)
**By region**						
North & South America (33)	27 (82%)	14 (42%)	17 (54%)	7 (21%)	2 (6%)	NA
Scandinavia (31)	24 (77%)	13 (42%)	21 (68%)	8 (26%)	6 (19%)	1 (3%)
Europe (excluding Scandinavia) (20)	18 (90%)	13 (65%)	9 (45%)	6 (30%)	2 (10%)	1 (5%)
Oceania (27)	14 (61%)	13(57%)	17 (74%)	9 (39%)	2 (7%)	NA
Global (8)	7 (88%)	3 (38%)	4 (50%)	2 (25%)	NA	NA
Asia (2)	2 (100%)	1 (50%)	1 (50%)	1 (50%)	NA	NA
**By condition**						
Arthroplasty/Reconstruction/Joint related registries (33)	29 (88%)	18 (55%)	21 (64%)	13 (40%)	7 (21%)	1 (3%)
Cancer registries (25)	18 (72%)	8 (32%)	17 (68%)	6 (24%)	3 (12%)	NA
Chronic disease registries (19)	14 (74%)	8 (42%)	8 (42%)	NA	1 (6%)	1 (6%)
Trauma/Burns/Pain registries (10)	6 (60%)	9 (90%)	6 (60%)	4 (40%)	NA	NA
Spine registries (8)	7 (88%)	5 (50%)	7 (88%)	3 (38%)	NA	NA
Miscellaneous registries (7)	7 (100%)	1 (14%)	1 (14%)	4 (50%)	NA	NA
Cardiac registries (6)	3 (50%)	3 (50%)	3 (50%)	NA	NA	NA
General surgery and device registries (5)	3 (60%)	3 (60%)	4 (80%)	3 (60%)	1 (20%)	NA
Rare disease registries (4)	2 (50%)	1 (25%)	NA	NA	NA	NA
Gynaecological registries (4)	3 (75%)	1 (25%)	2 (50%)	NA	NA	NA

When grouping the registries by health conditions, 29 (88%) Arthroplasty/Reconstruction/Joint related procedure registries captured PROMs at baseline followed by 18 (72%) Cancer registries. Eighteen (55%) Arthroplasty/Reconstruction/Joint related procedure registries collected PROMs at < 1 year follow up, followed by 9 (90%) Trauma/Burns/Pain, 8 (32%) Cancer and 8 (42%) Chronic disease registries.

### Average PROMs RRs

The overall mean and standard deviation (SD) RR of registries capturing PROMs started at 71% (24.0) at baseline and decreased to 56% (13.2) at 10 + years follow up period (Table 3).

**Table 3:** Average response rates (in %) with SD for time periods stratified by region, condition, modes and methods of administration and number of reminders sent. Follow up point t0 is the reported baseline time point or time of intervention as specified in the article or report. Follow up point t1 is from 0 to 1 year, follow up point t2 is from 1 to < 2 years, follow up point t3 is from 2 to < 5 years, follow up point t4 is from 5 to < 10 years and follow up point t5 is from 10 + years

	Follow up points					
	t0	t1	t2	t3	t4	t5
**All registries (n = 121)**	71 ± 24	65 ± 23	62 ± 20.5	59 ± 23.2	53 ± 15	56 ± 13.2
**By region**						
North & South America (33)	68 ± 26.7	60 ± 28.7	57 ± 22.5	63 ± 25.3	66 ± 13.3	N/A
Scandinavia (31)	73 ± 25.4	66 ± 21.0	66 ± 19.5	61 ± 16.4	51 ± 5.1	51 ± 10.7
Europe (excluding Scandinavia) (20)	74 ± 21.9	62 ± 24.0	65 ± 21.3	44 ± 32.9	53 ± 33.2	71 ± N/A
Oceania (27)	75 ± 17.8	72 ± 11.4	60 ± 18.2	63 ± 22.7	40 ± 2.5	N/A
Global (8)	54 ± 20.0	63 ± 35.9	51 ± 28.9	59 ± 7.7	N/A	N/A
Asia (2)	99 ± 2.1	89 ± N/A	92 ± N/A	72 ± N/A	N/A	N/A
**By condition**						
Arthroplasty/Reconstruction/Joint related registries (33)	68 ± 25.5	58 ± 22.3	61 ± 20.1	52 ± 27.6	51 ± 17.0	54 ± 15.5
Cancer registries (25)	75 ± 21.1	60 ± 33.3	61 ± 18.4	69 ± 16.0	59 ± 17.0	N/A
Chronic disease registries (19)	85 ± 15.1	73 ± 18.3	62 ± 25.4	70 ± 14.1	54 ± 6.9	61 ± N/A
Trauma/Burns/Pain registries (10)	69 ± 26.7	73 ± 15.6	65 ± 21.9	54 ± 32.3	N/A	N/A
Spine registries (8)	71 ± 19.8	73 ± 11.9	69 ± 16.9	66 ± 12	N/A	N/A
Miscellaneous registries (7)	70 ± 22.2	75 ± 24.2	41 ± N/A	N/A	N/A	N/A
Cardiac registries (6)	77 ± 34.9	59 ± 29.7	81 ± 12.8	N/A	N/A	N/A
General surgery and device registries (5)	35 ± 2.1	42 ± 14.9	45 ± 24.0	40 ± N/A	38 ± N/A	N/A
Rare diseases registries (4)	44 ± 34.3	88 ± 1.3	N/A	N/A	N/A	N/A
Gynaecological registries (4)	49 ± N/A	58 ± 46.0	60 ± 31.3	N/A	N/A	N/A
**Mode of administration**						
Electronic (25)	63 ± 25.7	59 ± 27.7	61 ± 26.7	50 ± 26.2	50 ± 2.1	NA
Paper (28)	73 ± 23.5	72 ± 20.3	61 ± 20.0	59 ± 19.4	57 ± 14.9	57 ± 6.6
Phone (5)	100 ± NA	69 ± 21.3	74 ± 32.2	81 ± NA	NA	NA
Mixed (47)	71 ± 23.6	85 ± 22.6	61 ± 20.7	54 ± 27.0	55 ± 21.9	55 ± 21.8
**Number of reminders sent**						
0 (24)	63 ± 28.0	56 ± 25.2	52 ± 23.7	50 ± 34.2	44 ± 20.2	61 ± NA
1 (22)	72 ± 17.0	76 ± 12.1	68 ± 15.3	65 ± 15.9	51 ± 11.1	46 ± 8.5
> 1 (17)	79 ± 18.5	62 ± 14.4	65 ± 19.3	53 ± 31.4	77 ± NA	71 ± NA

* If there is no SD the average consists of only one data point

**Fig. 2:**
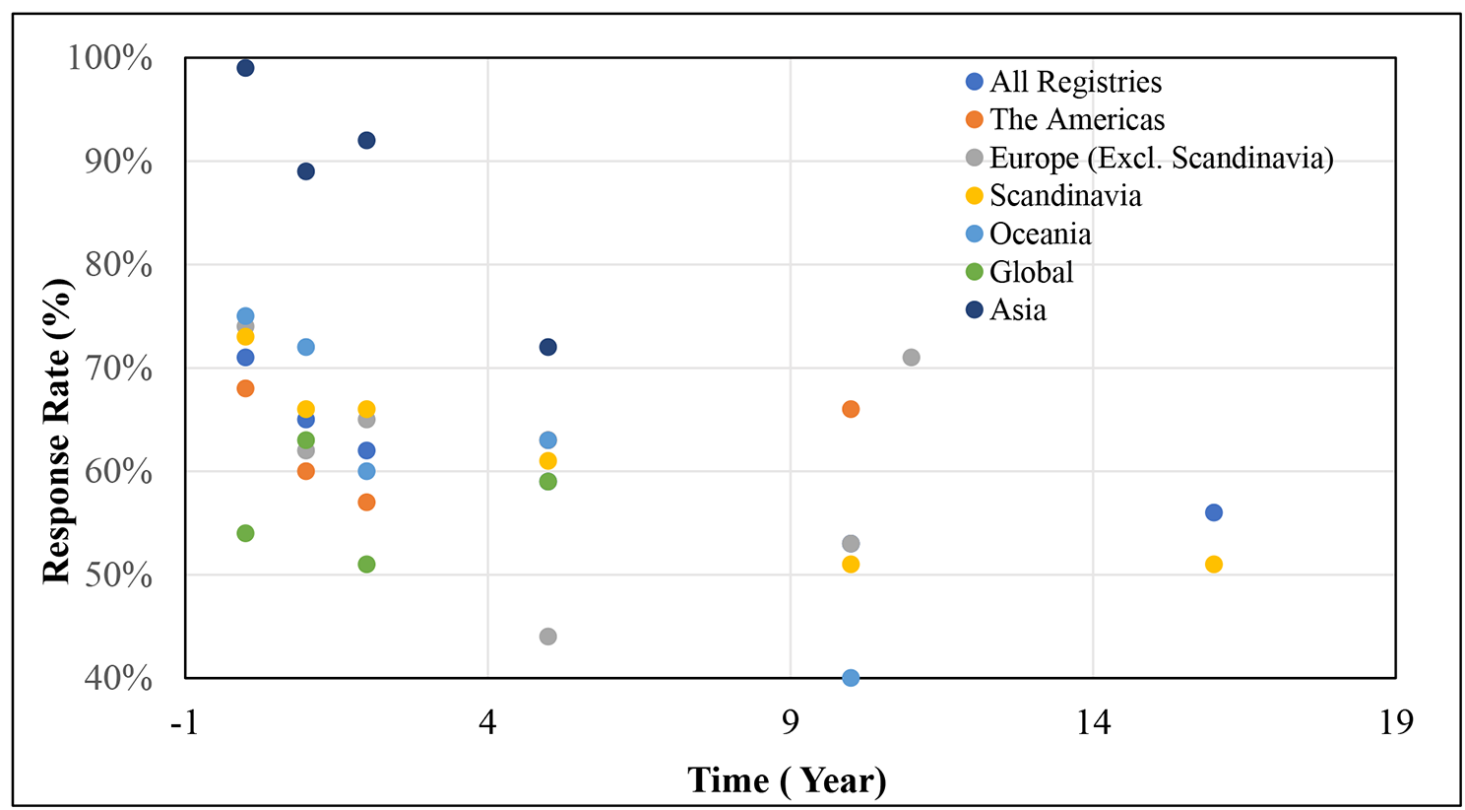
Average PROMs response rates over time according to regions

Disaggregating this data according to the regions of the world, the average PROMs RR decreased as follow up time period increased in most regions of the world except for the registries based in the North and South Americas, European (non-Scandinavian) registries and global registries. For North and South American registries, the average PROMs RR decreased until the 1 to < 2 years follow up mark, then increased in the subsequent years. The RRs for European and global registries increased and decreased alternatively at each time point. This trend is further illustrated in Fig. 2.

The highest average baseline RR of 99% was observed in Asian registries. In contrast, the lowest baseline RR of 54% was observed in global registries (Table 3).

**Fig. 3:**
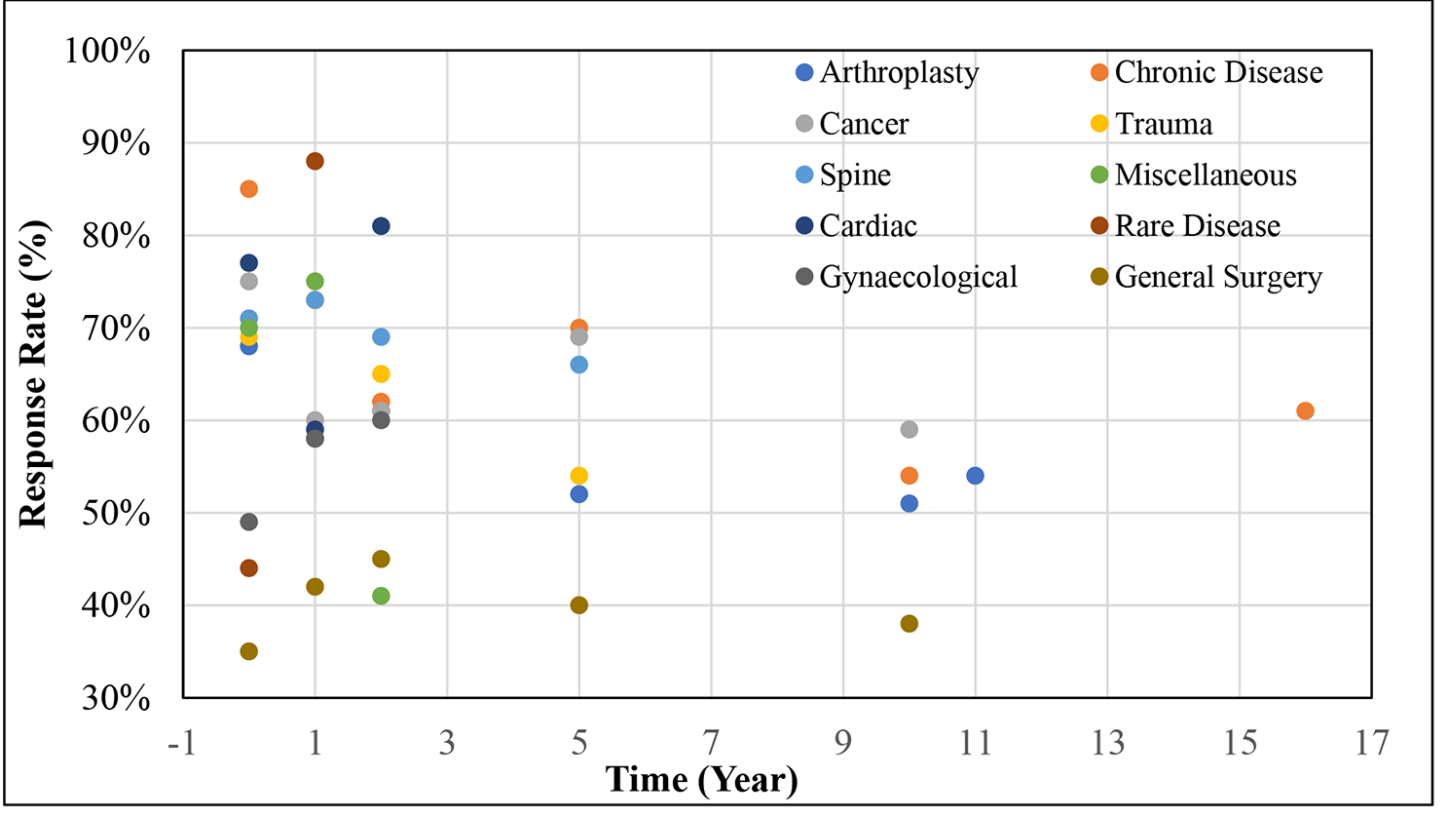
Average PROMs response rates over time according to health condition

When the data were disaggregated according to health conditions, all registries displayed varying trends as follow up years increased. The lowest baseline RR of 35% was reported by General surgery and device registries. Arthroplasty/Reconstruction/Joint related, Cancer and Cardiac registries exhibited a downward trend in RRs after baseline data collection, then increased in RRs at 1 to < 2 years follow up. In contrast, Trauma/Burns/Pain related, Spine and Miscellaneous registries displayed an increasing trend in RRs after baseline, and a decrease in RRs at 1 to < 2 years follow up period. Rare disease and Gynaecological registries exhibited an upward trend in RRs post baseline data collection. These trends are further illustrated in Fig. 3 displaying the average RRs categorized into health conditions.

PROMs data were collected for the longest follow up period of 10 + years by the Arthroplasty/Reconstruction/Joint related and Chronic disease registries. Cancer and General surgery and device registries reported PROMs data until the 5 to < 10 years follow up. Rare disease registries captured PROMs for the least amount of time [16–20], with the data being captured for less than a year.

At baseline, registries collecting PROMs on the phone reported the highest RR of 100%. This was followed by paper-based mode of administration (73%) and mixed method administration (71%). Some example include the American Registry for Migraine Research [21], the Expanded Haemodialysis Registry Protocol in Colombia [22] and the Prostate Strategic Urologic Research Endeavor Prostate Cancer Registry [23] which recorded nearly 100% RR at baseline. Registries using combined methods with nearly 100% baseline RR included the New Zealand Anterior Cruciate Ligament (ACL) Registry [24], the Keio inter-hospital Cardiovascular Studies-atrial fibrillation Registry [25] and Swedish Quality Registry for Pain Rehabilitation [26].

Electronic PROMs collection method was the least effective with an average baseline RR of 63% (25.7). Only the Austrian Myeloid Registry [27] and the Outcomes Registry for Better Informed Treatment of Atrial Fibrillation [28] recorded the highest RR at baseline (99% and 94% respectively).

Registries that sent more than one reminder led to a higher RR at baseline of 79% compared to those sending no reminders (63%) or only one reminder (72%) (Table 3). Those with more than one reminder recorded PROMs RR over 98%. Examples include Prostate Cancer Outcomes Registry-Victoria [29] and the New Zealand ACL Registry [13] both obtaining baseline RR over 98%. There was no identifiable trend in RRs in registries that sent more than one, one or no reminders for PROMs as follow up years increased.

### Change in RR over time

**Fig. 4:**
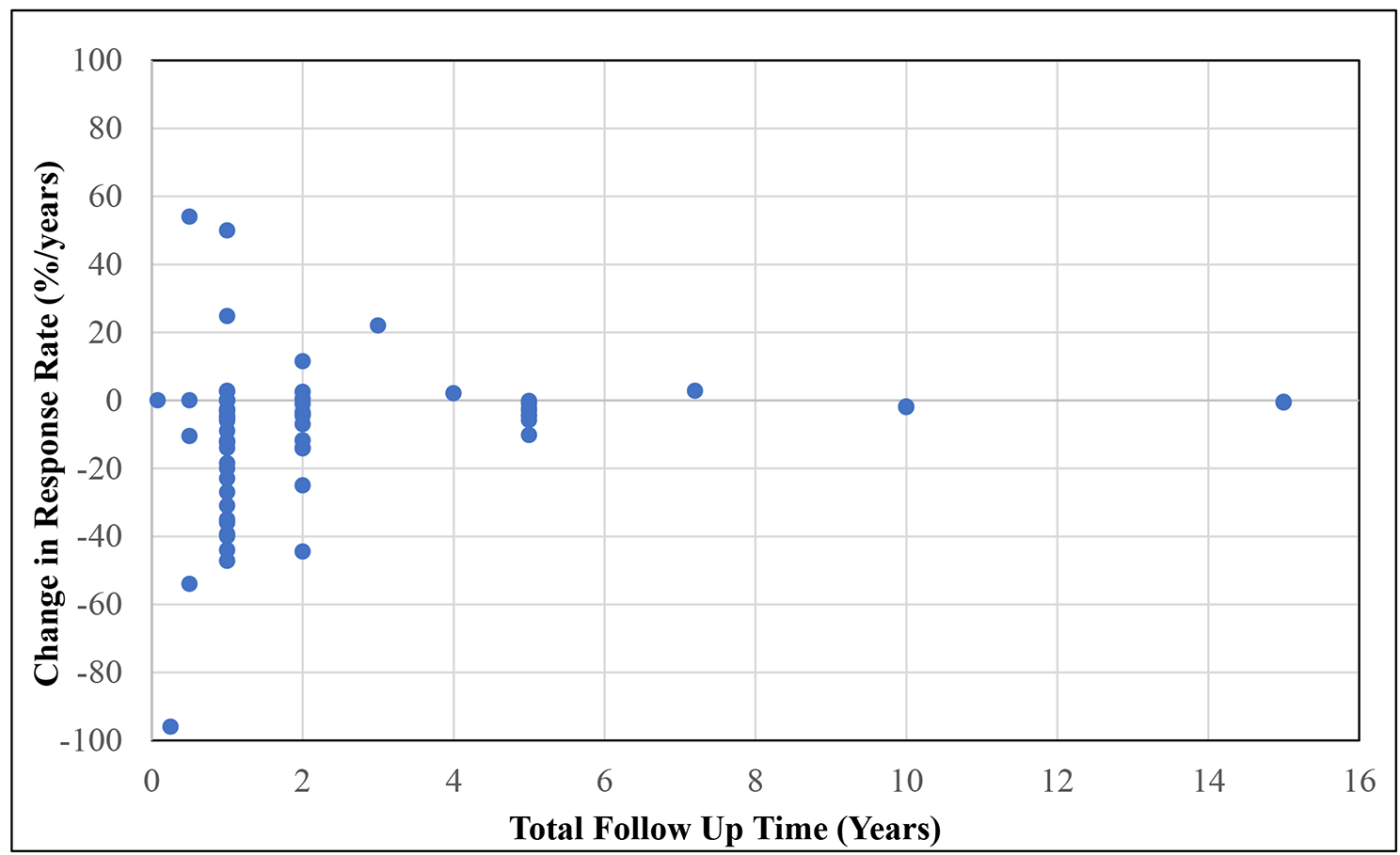
Change in PROMs response rates over total follow up time. In this figure the change in response rates and total follow up time point for the Netherlands Cancer Registry was not plotted. This was due to the registry collecting follow up data seven days post treatment, hence once the change in response rates was calculated, the number is a large outlier that goes beyond the scale of the figure

Figure 4 portrays the change in RR over time according to the total follow up years of PROMs data capture. Of the 121 registries identified in our search, 54 registries captured PROMs only once. Change in RR over time could not be calculated for these registries. Change in RR approached to zero as the total follow up time increased indicating smaller change in RRs for 67 registries as follow up time increased.

## Discussion

This is an up to date scoping review which aimed to describe RRs of PROMs captured in clinical registries and databases at various follow up timepoints. This review has identified 121 registries and databases capturing PROMs over at least one time point. Most of these registries were based in North and South Americas and Scandinavia, and captured PROMs at five different time points for ten or more years.

The overall average baseline RR for the registries included in this study was 71%, similar to that of 75% reported by Wang et al. [13]. As expected, the RR trended down over time, but with a slight increase of RR after ten years of follow up.

The highest baseline RR was observed in Chronic (85%) and Cancer (75%) disease registries. This could possibly occur due to symptom burden and reduced HRQoL in patients suffering from cancer and/or chronic illnesses. In general, chronic diseases are slow in progression, long in duration and also require regular medical monitoring and treatment [30]. Such conditions include stroke, diabetes, bowel disease, renal disease and diseases of the central nervous system and are associated with poor HRQoL. Since the attention is turning to patients with chronic conditions, PROMs can be used to provide patients’ perspective about impacts on their health status based on the choice of drug therapy and care provider. Care for such patients and their HRQoL might be improved if registries monitor PROMs routinely over a longer period of time [31].

Of 121 registries identified in this review, Arthroplasty/Reconstruction/Joint related registries were most common. This is not surprising, as the older population is growing in number and older adults are living longer. With fragility fractures and other fall-related injuries negatively impacting their HRQoL, limiting autonomy and increasing disability, they often require various joint and hip reconstruction procedures [30]. Such registries monitor patients for a long time and therefore, it seems reasonable that they capture PROMs at various follow up time points for more than ten years, with the RR varying from 68% at baseline to mid-50% at ten years post-surgery.

With regard to the number of PROMs reminders, our results reflect conclusions from previous studies confirming that more than one reminder is required to improve RRs [13, 32]. A similar study by Lucas et al. [33] was designed to capture electronic PROMs in prostate cancer patients. A systematic method that included automated email reminders, by which repeat contact was structured within the survey process, resulted in relatively high PROMs RRs at baseline and follow up.

PROMs delivery method and mode of administration need not to be ignored either. Studies have already shown that focusing on digital mechanisms, such as email and SMS, can achieve up to 97% RRs [34]. It also appears that postal mode of PROMs administration seems to perform better than electronic means but it can be more time-consuming and resource-intensive as the data needs to be digitized afterwards [35].

The benefits of PROMs are widely accepted; however, achieving high RRs remains a significant barrier and can be influenced by many different factors. To achieve goals of evaluating treatments and improving patient care, a certain RR to PROMs is necessary [24]. The International Society of Arthroplasty Registries PROMs Working Group proposed a RR of at least 60% [25]. This number is based on what is considered a sufficient RR in survey research [26]. Unfortunately, there is no clear consensus of what RR is acceptable for other registries so far.

Achieving high RRs at multiple follow up data collection points is challenging [24]. A recently published study by Ho et al. [36] assessed predictors of successful PROMs RRs in an orthopaedic outpatient setting at a public tertiary hospital. Being younger, being a new patient, having a longer wait time, being an English-speaker and being a pre- or post-operative patient were all associated with an increased RR of PROMs in this study. A similar study of 205 medical and surgical hospitals evaluated both patient and clinician factors in regards to RR to PROMs [37]. The factors included clinician training for PROMs data collection, administrative oversight, previous experience, presence of a clinician champion and payer incentives. Most of these factors were tied to a better RR. Just about half of all clinics studied yielded a 50% PROMs collection rate or better. Overwhelmingly, a high PROMs RR was linked to having at least 50% of clinicians trained in collecting patient responses and having administrative leaders oversee the whole process. Having prior experience with paper-based PROMs collection was also important [37].

Actions to improve RRs in clinical registries are needed. These may include capturing shorter forms of questionnaires or offering proxy versions for those who are ill or unable to complete the forms themselves [38, 39]. Translated in different languages and culturally-adapted versions of PROMs for non-native speakers should be also considered. PROMs data should be regularly discussed with patients and at consumer forums to encourage more adherence, which can possibly lead to improved RRs and better-quality of the data [40].

### Strengths and limitations

In this study, we comprehensively reviewed a large number of clinical registries and databases from all over the world, with comparisons made across different regions and health conditions. To appreciate the findings in this review, the following limitations should be considered. First, we have likely missed several registries and databases despite our comprehensive search strategy, including an internet search in addition to a literature search of main large electronic databases. Second, a few publications and grey literature sources did not provide detailed information on the RRs or follow up time points. This has been noted in the text and tables. Third, some of the RRs in this review were extracted from registry cohort studies and may not reflect the actual RR at particular follow up points.

## Conclusions

This review demonstrated large variation and downward trends of RRs to PROMs captured in clinical registries and databases across world regions and various health conditions. We have demonstrated that RRs to PROMs in a registry setting are constantly changing as they can be influenced by many amendable factors. Guidelines and recommendations for PROMs inclusion and capture in clinical registries should be considered prior to determining timing, frequency, mode and method for PROMs administration [6]. To date, there is no clear evidence for acceptable RR to PROMs in clinical registries. Consequently, further studies are warranted to determine reasonable RRs to PROMs while maintaining collection of high-quality clinical and patient outcome data.

## Electronic supplementary material

Below is the link to the electronic supplementary material.

**Additional file 1**. Provides the search strategy used in EMBASE and MEDLINE.

## Data Availability

Full data extraction table available upon request. Summary of results in Table 1.

## Abbreviations

HRQoL: Health Related Quality of Life
MeSH: Medical Subject Heading
PRISMA-ScR: Preferred Reporting Items for Systematic Reviews and Meta-Analyses extension for scoping reviews
PROMs: Patient Reported Outcome Measures
RR: Response Rates
SD: Standard Deviation
