# Supplementary material for: Response rates in clinical quality registries and databases that collect patient reported outcome measures: a scoping review
Source: Health Qual Life Outcomes. 2023 Jul 11;21:71. doi: 10.1186/s12955-023-02155-5 (PMC10337187; doi:10.1186/s12955-023-02155-5)
Supplement: Supplementary file 1 — Additional file 1. Provides the search strategy used in EMBASE and MEDLINE. [file 12955_2023_2155_MOESM1_ESM.docx]

Additional file 1: Terms used in the search

| **EMBASE Search** | **MEDLINE Search** |
| --- | --- |
| 1. register/ or patient registry/ 2. (register* or registr*).mp. 3. 1 or 2 4. patient-reported outcome/ 5. (prom* adj1 registr*).mp. 6. (patient report* outcome* measure* adj4 registr*).mp. 7. ((patient reported outcomes or patient-reported outcomes or patient reported outcome measures or patient-reported outcome measures or PROMs) adj3 (register* or registr*)).mp. 8. 4 or 5 or 6 or 7 9. 3 and 8 10. questionnaire/ 11. (survey* or questionnair*).mp 12. 10 or 11 13. 9 and 12 14. limit 13 to english language 15. limit 14 to (conference abstracts or medline) 16. 14 not 15 17. limit 16 to (books or chapter or conference abstract or editorial or letter or note or short survey or tombstone) 18. 16 not 17 19. limit 18 to (book or book series or conference proceeding or trade journal) 20. 18 not 19 21. 20 not (address or autobiography or bibliography or biography or case report* or conference or comment or congress or consensus development conference or dictionary or directory or editorial or festschrift or guideline or historical article or interactive tutorial or interview or lecture or legal case or legislation or letter or news or newspaper article or patient education handout* or periodical index or personal narrative or portrait or practice guideline or veterinar* or video-audio media or webcast).mp. 22. 21 not (newspaper* or Anecdote* or Biograph* or Book or Book Chapter* or Book Review* or, Brief Item* or Case Study or Directories or Dissertation or Thesis or Theses or Exam Questions or Pamphlet or Proceedings).af. | 1. Registries/ 2. (register* or registr*).mp. 3. 1 or 2 4. patient reported outcome measures/ 5. (prom* adj1 registr*).mp. 6. (patient report* outcome* measur* adj4 registr*).mp. 7. ((patient reported outcomes or patient-reported outcomes or patient reported outcome measures or patient-reported outcome measures or PROMs) adj3 (register* or registr*)).mp. 8. 4 or 5 or 6 or 7 9. 3 and 8 10. "Surveys and Questionnaires"/ 11. (questionnaire* or survey*).mp. 12. 10 or 11 13. 9 and 12 14. limit 13 to english language 15. Limit 14 to (address or autobiography or bibliography or biography or clinical conference or clinical trial, veterinary or clinical trial protocol or congress or consensus development conference or consensus development conference, nih or dataset or dictionary or directory or duplicate publication or editorial or English abstract or electronic supplementary materials or “Expression of concern" or festschrift or guideline or historical article or interactive tutorial or interview or lecture or legal case or legislation or letter or news or newspaper article or observational study, veterinary or patient education handout or periodical index or personal narrative or portrait or practice guideline or randomized controlled trial, veterinary or video-audio media or webcast) 16. 14 not 15 17. 16 not (address or autobiography or bibliography or biography or case report* or conference or comment or congress or consensus development conference or dictionary or directory or editorial or festschrift or guideline or historical article or interactive tutorial or interview or lecture or legal case or legislation or letter or news or newspaper article or patient education handout* or periodical index or personal narrative or portrait or practice guideline or veterinar* or video-audio media or webcast).mp. 18. 17 not (newspaper* or anecdote* or biograph* or book or book chapter* or book review* or, brief item* or case study or directories or dissertation or thesis or theses or exam questions or pamphlet or proceedings).af. |
